# Exploring the Therapeutic Potentials of Exopolysaccharides Derived From Lactic Acid Bacteria and Bifidobacteria: Antioxidant, Antitumor, and Periodontal Regeneration

**DOI:** 10.3389/fmicb.2022.803688

**Published:** 2022-04-25

**Authors:** Maha A. Khalil, Fatma I. Sonbol, Lamiaa A. Al-Madboly, Tamer A. Aboshady, Abeer S. Alqurashi, Sameh S. Ali

**Affiliations:** ^1^Biology Department, College of Science, Taif University, Taif, Saudi Arabia; ^2^Botany and Microbiology Department, Faculty of Science, Tanta University, Tanta, Egypt; ^3^Pharmaceutical Microbiology Department, Faculty of Pharmacy, Tanta University, Tanta, Egypt; ^4^Periodontology, Oral Medicine, Diagnosis and Radiology Department, Faculty of Dentistry, Tanta University, Tanta, Egypt; ^5^Oral and Maxillofacial Surgery and Diagnostic Sciences, Faculty of Dentistry, Taif University, Taif, Saudi Arabia; ^6^Biofuels Institute, School of the Environment and Safety Engineering, Jiangsu University, Zhenjiang, China

**Keywords:** lactic acid bacteria, bifidobacteria, exopolysaccharide, RT-PCR, MCF7, CaCo2, HepG2, therapeutic agent

## Abstract

The metabolites of lactic acid bacteria (LAB) and bifidobacteria (Bb) have recently received a lot of attention due to their ability to protect interactions in blood and tissues, as well as their biodegradability and biocompatibility in human tissue. Exopolysaccharides (EPS) derived from bacteria have a long history of use in therapeutic and other industrial applications with no adverse effects. In this regard, EPSs were isolated and characterized from LAB and Bb culture supernatants to determine their antioxidant, antitumor, and periodontal regeneration properties. The antioxidant capacity of the EPSs varied with concentration (0.625–20 mg/ml). The highest antioxidant activity was found in LAB: *Streptococcus thermophiles* DSM 24731-EPS_1_, *Lactobacillus delbrueckii* ssp. *bulgaricus* DSM 20081^T^-EPS_5_, *Limosilactobacillus fermentum* DSM 20049-EPS_6_, and Bb; *Bifidobacterium longum* ssp. *longum* DSM 200707-EPS_10_. Human breast cancer cells (MCF7), human colon cancer cells (CaCo2), human liver cancer cells (HepG2), and human embryonic kidney 293 (HEK 293) cells were used as controls to assess the antitumor properties of the selected EPSs. According to the 3-(4,5-dimethylthiazol-2-yl)-2,5-diphenyltetrazolium-bromide (MTT) assay, EPS_5_ had the highest cytotoxicity against MCF7, CaCo2, and HepG2, with IC_50_ values of 7.91, 10.69, and 9.12 mg/ml, respectively. Lactate dehydrogenase (LDH) activity was significantly higher in cell lines treated with EPS_5_-IC_50_ values compared to other EPSs-IC_50_ values (*p* < 0.05). Real time (RT)-PCR results showed that EPS_5_ treatment increased *Bax*, *Caspase 8*, *Caspase 3*, and *p53* gene expression. The expression of the *BCL2*, *MCL1*, and *Vimentin* genes, on the other hand, was reduced. The MTT test was used to examine the effect of EPS_5_ on the viability of human periodontal ligament fibroblast cells (hPDLFCs), and it was discovered that EPS_5_ increased hPDLFC viability. According to high-performance liquid chromatography (HPLC) analysis, galactose made up 12.5% of EPS_5_. The findings of this study pave the way for the use of EPS, which hold great promise for a variety of therapeutic purposes such as antioxidant, antitumor, and periodontal regeneration.

## Graphical Abstract

Therapeutic applications of exopolysaccharides derived from lactic acid bacteria and bifidobacteria.

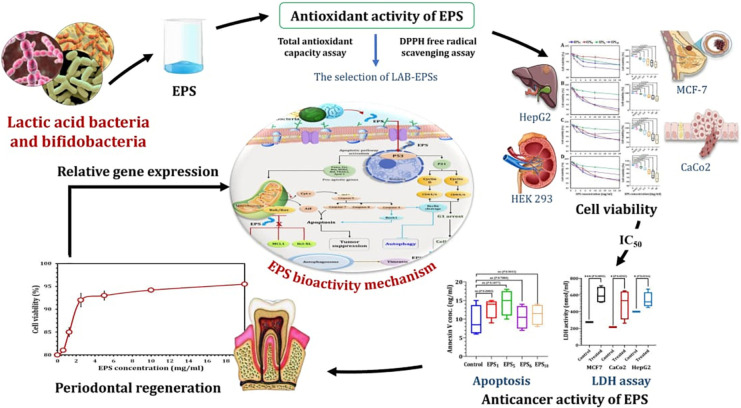

## Introduction

Bacteria are recognized for their ability to produce a wide variety of polysaccharides. These polysaccharides may either be tightly attached to the cell surface as capsular polysaccharides (CPS) or they can be released as exopolysaccharides (EPSs). Many bacterial taxa, particularly lactic acid bacteria (LAB) and bifidobacteria (Bb), produce a wide range of carbohydrate polymers during fermentation (Sanalibaba and Cakmak, 2016). LAB are generally considered as safe microorganisms (GRAS—generally recognized as safe) and also capable of creating EPSs with many different structures without any health risks (Surayot et al., 2014). LAB refers to Gram-positive bacteria that are often isolated from fermented natural products and are extensively employed in industrial operations. LAB and their metabolic products have been shown to enhance immunity, gastro-intestinal function, resistance to obesity, antioxidant activity, and blood glucose and cholesterol levels (Mathur et al., 2020; Wang et al., 2020). Additionally, they also have potential health benefits such as anticancer activity (Tukenmez et al., 2019), immunostimulatory activity (Adebayo-Tayo et al., 2018), antibiofilm activity (Lakra et al., 2020), and antiviral activity (Biliavska et al., 2019).

Exopolysaccharides have garnered significant interest in recent years owing to their potential pharmacological and biomedical uses (Barcelos et al., 2019). EPSs are classified into two categories: homopolysaccharides (HoPs) are composed entirely of single monosaccharides, heteropolysaccharides (HePs) are composed of a polymer chain of monomer units composed of two or more monosaccharides (Mende et al., 2016). High-molecular-mass polysaccharides exhibit a variety of chemical properties, including molecular weights, charges, side chains, and rigidity (Sanalibaba and Cakmak, 2016; Wang et al., 2020). Simultaneously, the growth conditions used to produce probiotic bacteria alter the chemical structure of EPS (Zannini et al., 2016). Additionally, experiments have demonstrated that the composition and structure of EPSs are strain-dependent (Li C. et al., 2014). Due to their chemical properties, including molecular structures, chain linkage, and molecular mass, EPS exhibit anticancer activity with minimal side effects. As a result, EPS with a diverse chemical structure and property profile may be advantageous in a variety of therapeutic applications (Wang et al., 2014; Hussain et al., 2017; Lin et al., 2018).

Concerns have grown about the safety and toxicity of synthetic antioxidants, but researchers face a difficult task in identifying natural antioxidants that are safe for human health (Adebayo-Tayo and Fashogbon, 2020). It has recently been demonstrated that LAB EPS have antioxidant properties and could be used as potential antioxidants (El-Adawi et al., 2012; Adebayo-Tayo et al., 2018). *Lactobacillus helveticus* MB2-1 EPS has significant antioxidant and anticancer activity in colon cancer cells (Li W. et al., 2014; Li et al., 2015). Adebayo-Tayo and Fashogbon (2020) confirmed that EPS activity has pharmacological and nutraceutical applications in wild-type and mutant *Lactobacillus delbrueckii*. Furthermore, Joo Seo et al. (2015) established that purified *Lactiplantibacillus plantarum* YML009-EPS had high antioxidant activity. *Lactobacillus acidophilus*, *Lactobacillus gasseri*, *Lactiplantibacillus plantarum*, and *Lacticaseibacillus rhamnosus* were reported to have anticancer and antioxidant properties (Sungur et al., 2017; Adesulu-Dahunsi et al., 2018). The antioxidant activity of EPS extracted from fermented milk of *L. delbrueckii* ssp. *bulgaricus* SRFM-1 has been demonstrated to be acceptable (Tang et al., 2017). Additionally, EPS extracted from *L. plantarum* ZDY2013 was shown to have increased antioxidant activity following sulfonation (Zhang et al., 2016).

Periodontal diseases are noteworthy as a global “hidden” epidemic in terms of disease burden and socioeconomic consequences. Periodontal disease is caused by bacteria that live in the gingiva and on the tooth and is characterized by inflammation and destruction of the oral supporting tissue. If the biofilm is not removed from the periodontal pocket, it may induce an immune response and inflammation in other periodontal tissues, resulting in progressive periodontitis. Periodontitis left untreated may result in further bone loss and, eventually, tooth loss (Nasajpour et al., 2018). While dental scaling and open-flap surgery are effective at removing biofilms and regulating inflammation in order to prevent disease development, they are ineffective at regenerating periodontal tissue (Iviglia et al., 2019). The identification of naturally occurring biomaterials with multiple functions may hold enormous promise for periodontal repair and regeneration, as well as serve as a springboard for future research.

At the moment, researchers are investigating low-risk cancer medications, and one of their areas of interest is the antitumor properties of LAB (Norouzi et al., 2018). Cancer is a term that refers to the uncontrolled proliferation of abnormal cells that have the ability to invade and damage surrounding tissue. According to the Global Cancer Center, cancer has surpassed heart disease as the leading cause of death (Bray et al., 2018). Surgical excision, radiation, and medication therapy are all considered traditional cancer therapies (Qin, 2015). Drug therapy can have a detrimental effect on normal organs and tissues, including the bone marrow, kidneys, and oral mucosa, as well as impair normal metabolism. Additionally, these three approaches may result in inflammation and subsequent lymphedema following therapy (Hu et al., 2017; Adebayo-Tayo and Fashogbon, 2020). As a result of their affordability, availability, and lack of adverse effects, people prefer complementary and alternative medications (Liu et al., 2019). As a result, there is an increasing need for targeted therapies capable of successfully treating cancer while having a negligible effect on normal tissues or at the very least acting as adjuvants to reduce clinical doses and increase the potency of conventional chemotherapy agents (Liu et al., 2021).

Lactobacilli are anticancer agents because they can induce apoptosis in cancerous cells, differentiate them, and bind to genotoxic substances (Wu et al., 2021). Due to the fact that LAB EPS inhibits the growth of a variety of tumor cells, including those from the stomach, liver, and breast, it may be used as a complementary or alternative cancer treatment. These agents have a variety of antitumor effects, including cell cycle arrest and inducing apoptosis, as well as antimutagenic, antioxidative, antiangiogenesis, and anti-inflammatory properties (Nazir et al., 2018; Milner et al., 2021; Wu et al., 2021). Additionally, EPS has a wide range of effects, including decreasing tumor-promoting enzyme activity, increasing immune response, forming host-supporting metabolites and resistant pathogens, and limiting cell proliferation by inhibiting cancer cell spread *via* apoptosis (Liu et al., 2021). Apoptosis (programmed cell death) occurs at the molecular level *via* two distinct mechanisms, intrinsic and extrinsic (Śliżewska et al., 2021). The *BCl2* family of pro- and anti-apoptotic proteins regulates intrinsic or mitochondrial pathways. Exogenous apoptosis, alternatively referred to as the cytoplasmic route, is mediated by the death receptor Fas, a member of the tumor necrosis factor family (Zhao et al., 2019). Numerous publications have demonstrated that EPSs possess a variety of biologically active properties, including antioxidant, immune-stimulating, and anticancer properties (El-Adawi et al., 2012; El-Deeb et al., 2018; Angelin and Kavitha, 2020). In this regard, EPSs from the marine bacterium *L. plantarum* 70810 have shown an anti-proliferative effect on a hepatocellular cancer cell line (HepG2) (Yahya et al., 2019). Rajoka et al. (2018) investigated the bioactivity of the cell-free culture supernatant (CFCS) of *Lactobacillus* strains *Lactobacillus casei* SR1, *L. casei* SR2, and *Lactobacillus paracasei* SR4 obtained from human breast milk. They revealed that the CFCSs exhibited adequate anticancer effects on cervical cancer (HeLa) cells by upregulating the apoptotic genes *BAX*, *BAD*, *Caspase 3*, *Caspase 8*, and *Caspase 9* and downregulating the *BCl2* gene expression.

In terms of EPS’s unique properties and potential effects, we hypothesized that it has a profound effect on periodontal repair and regeneration, as well as on cancer cell growth inhibition and apoptosis induction. To address this issue, we assessed the EPS’s cytotoxicity against breast cancer (MCF7), colon cancer (CaCo2), human liver cancer cells (HepG2), and human embryonic kidney 293 (HEK 293) cells (control), as well as its antioxidant capacity. Additionally, using a variety of cancer cell lines, the current research examined their effect on apoptosis-related gene expression. Additionally, the effect of EPS on the survival of human periodontal ligament fibroblast cells (hPDLFCs) *in vitro* was investigated.

Numerous research were performed recently to characterize and comprehend the biotechnological application of EPS produced from LAB and Bb. However, the tremendous therapeutic potential of EPS, particularly in periodontal healing and regeneration, is still unknown. Due to the wide range of uses for EPS and the rising demand for biopolymers, it is vital to develop new EPS produced from LAB. To our knowledge, the multifunctional therapeutic applications of EPS produced by *L. delbrueckii* ssp. *bulgaricus* DSM 20081^T^ have been emphasized for the first time. Under this scope, this work aims to look into the antioxidant, antitumor, and periodontal regeneration properties of EPSs derived using LAB and Bb culture supernatants. As a result, EPSs derived from *L. delbrueckii* ssp. *bulgaricus* DSM 20081^T^ may be a promising candidate for a variety of therapeutic applications.

## Materials and Methods

### Cell Culture

On Dulbecco’s Modified Eagle Medium (DMEM), non-cancerous HEK 293 cells were grown. CaCo2 and HepG2 cells were grown in DMEM; MCF7 cells were grown in Rosewell Park Memorial Institute (RPMI) medium. All cells were cultured in a medium containing 2 mM L-glutamine, 10% fetal bovine serum (FBS), and 1% penicillin–streptomycin combination. The cultures were incubated at 37°C for 24 h in a humidified environment containing 5% CO_2_ and 95% air.

### Bacterial Strains

#### Lactic Acid Bacteria

Lactic acid bacteria (n = 9) including *Streptococcus thermophiles* DSM 24731, *Lactobacillus lactis* ssp. *cremoris* DSM 20069^T^, *Lacticaseibacillus casei* DSM 27537, *Lactobacillus delbrueckii* ssp. *bulgaricus* DSM 20080, *Lactobacillus delbrueckii* ssp. *bulgaricus* DSM 20081^T^, *Limosilactobacillus fermentum* DSM 20049, *Lactobacillus acidophilus* DSM 20079^T^, *Lactobacillus rhamnosus* DSM 20021, and *Lactobacillus plantarum* ssp. *plantarum* DSM 20174 were kindly obtained from the culture collection of the Faculty of Agriculture Ain Shams University and the Faculty of Science Tanta University (El-Adawi et al., 2012). LAB strains were grown in De Man–Rogosa–Sharpe (MRS, Biokar Diagnostics, Beauvais, France) broth [10.0 g/l peptone, 8.0 g/l meat extract, 4.0 g/l yeast extract, 20.0 g/l D (+)-glucose, 2.0 g/l dipotassium hydrogen phosphate, 5 g/l sodium acetate trihydrate, 2.0 g/l triammonium citrate, 0.2 g/l magnesium sulfate heptahydrate, 0.05 g/l manganous sulfate tetrahydrate, final pH 6.2] at 37°C for 24 h anaerobically. However, *Streptococcus thermophilus*, was cultured on M17 agar media and incubated at 37°C under aerobic and anaerobic conditions.

#### Bifidobacterial Strain

*Bifidobacterium longum* ssp. *longum* DSM 200707 was provided from Kafr El-Sheik University. *Bifidobacterium* was grown in MRS medium supplemented with l-cysteine at 0.5 g/l (MRSc) at 37°C in anaerobic environment.

### Extraction of Crude Exopolysaccharides

**FIGURE 1:**
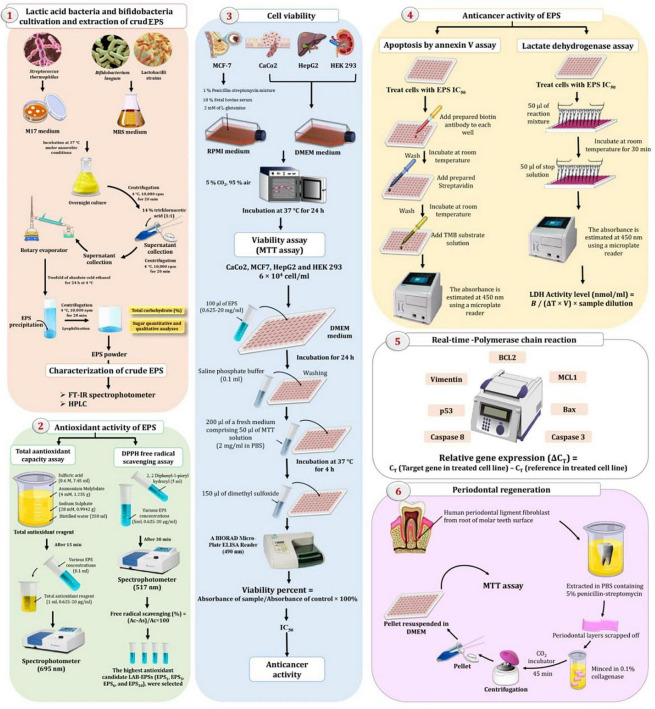
Experimental setup for extraction and characterization of exopolysaccharides (EPS) by lactic acid bacteria (LAB) and bifidobacteria (Bb) exploring their therapeutic potentials.

The experimental setup for extracting EPS from a variety of LAB is depicted in Figure 1. Centrifugation at 4°C, 10,000 rpm, for approximately 20 min was used to remove bacteria from overnight cultures (Bajpai et al., 2016). The supernatant was collected and diluted with 14% trichloroacetic acid (1:1), followed by centrifugation (10,000 rpm at 4°C for 20 min) to remove the proteins. The clear supernatant was collected and concentrated using a rotary evaporator. The EPS was precipitated by adding double the amount of absolute cold ethanol and maintaining it at 4°C for 24 h, followed by 20 min of centrifugation at 4°C, 10,000 rpm. After dilution with deionized water, the residue was dialyzed for 48 h. The solution was concentrated and lyophilized to obtain dry powdered crude EPS (mg/l). The total carbohydrate content (%) of crude EPS was determined using the phenol-sulfuric acid method, using glucose (2 mg/ml) as a standard (Dubois et al., 1956). Approximately 0.5 ml of EPS solution (100 mg/ml) was combined with 0.5 ml of phenol 6% (v/v) in a test tube. A mixed solution of concentrated sulfuric acid (2.5 ml) was added to the test tube. After 10 min, the mixture was kept in a water bath (30°C) for 20 min, and the absorbance (Abs) at 490 nm was determined using a spectrophotometer. By substituting Abs into the standard curve, the carbohydrate content was determined. Simultaneously, the protein content of EPS was determined using Bradford method (Bradford, 1976). Protein reagent (2.5 ml, 50 ml containing 2.0 mg/ml Coomassie Brilliant Blue G-250 in 95% ethanol and 100 ml 85% phosphoric acid) was added to the polysaccharide solution (0.5 ml, 2 mg/ml). Abs at 595 nm was determined using a spectrophotometer following agitation. The protein content was determined by substituting Abs into a standard curve.

### Antioxidant Activity of Crude Exopolysaccharides

The 1,1-diphenyl-2-pyridyl-hydrazine (DPPH) assay was used to investigate the antioxidant activity of EPSs (Kao and Chen, 2006) and the total antioxidant capacity (TAC) (Dilna et al., 2015), as shown in Figure 1.

#### 1,1-Diphenyl-2-Pyridyl-Hydrazine Free Radical Scavenging Assay

The ability of EPS to scavenge DPPH radicals was determined using the procedures described by Kao and Chen (2006). Five millimeters of 0.02 methanolic DPPH radical solution was added to 5 ml of various EPS concentrations (0.625–20 mg/ml). For 30 min, the reaction mixture was kept in a darkroom. The Abs of the mixture was determined spectrophotometrically at 517 nm using a Spectrophotometer UV-visible 2401PC (Shimadzu, Japan). Three times for each measurement, the means and standard deviations (SD) were computed. The following equation was used to determine the capacity to scavenge the DPPH radical:


Freeradicalscavenging%=[1-(Ac-As)/Ac]×100.

Ac, Absorbance of control (all reagents except the checked substance is replaced by distilled water).

As, Absorbance of the sample/standard.

Ascorbic acid has traditionally been utilized as a free radical scavenger.

#### Total Antioxidant Capacity Assay

The TAC of the EPS samples was determined as described by Dilna et al. (2015). To make the TAC reagent, mix 7.45 ml sulfuric acid (0.6 M), 1.235 g ammonium molybdate (4 mM), and 0.9942 g sodium sulfate (28 mM) in 250 ml distilled water. After 15 min, 0.1 ml of various EPS concentrations (0.625–20 mg/ml) were dissolved in 1 ml of total antioxidant reagent, and the Abs at 695 nm was measured with a spectrophotometer. Ascorbic acid served as a control substance. Additionally, the most antioxidant-potent candidate EPSs (EPS_1_, EPS_5_, EPS_6_, and EPS_10_) were chosen for future investigation.

### Cytotoxicity Assay

The 3-(4,5-dimethylthiazol-2-yl)-2,5-diphenyltetrazolium-bromide (MTT) test was used to assess cytotoxicity (Sigma Aldrich, St. Louis, MO, United States), as previously described by Manivasagan et al. (2013) and Khalil et al. (2022). The cells (100 μl, MCF7, CaCo2, and HepG2) were seeded at a density of 6 × 10^4^ cells/ml in 96-well microplates and treated with EPS_1_, EPS_5_, EPS_6_, and EPS_10_ at various concentrations (0.625–20 mg/ml). The optical density of the formazan solution was determined using a BIORAD Micro-Plate ELISA Reader at 490 nm. Additionally, to ensure that the extracted EPSs are safe for normal cells, their inhibitory effects on the normal HEK 293 cell line were determined. Because MTT decreases only in metabolically active cells, activity is a proxy for cell viability. The EPS extracts were tested in triplicate on the five cell lines, and the viability of the cells was estimated using the following equation: Viability % = Absorbance of sample/Absorbance of control × 100. According to El-Deeb et al. (2018) and Khalil et al. (2022), the IC_50_ values for EPS were calculated using the graphpad prism 9 program and indicate a 50% reduction in growth when compared to control cells (untreated cells).

### Antitumor Activity

The annexin V test and the amount of lactate dehydrogenase (LDH) leaking out of the cell were used to determine the antitumor activity of EPSs (Gurunathan et al., 2013), as represented in Figure 1.

### Apoptosis Assessment

According to the manufacturer’s instructions, all cell lines exhibited apoptosis as determined by a human Annexin V platinum ELISA (eBioscience BMS252/BMS252TEN; Thermo Fisher Scientific, Waltham, MA, United States). For 24 h in a humidified incubator at 37°C and 5% CO_2_, cell lines were grown in 96-well flat-bottom culture plates with the IC_50_ concentrations of EPS_1_, EPS_5_, EPS_6_, and EPS_10_. This assay is based on the fact that human Annexin V present in the sample or standard will bind to the Microwell Plate coating antibodies. As a result, a biotin-conjugated anti-human Annexin V antibody was added, which specifically recognizes the human Annexin V captured by the first antibody. Following incubation, all unbound biotin-conjugated anti-human Annexin V antibody was removed during the washing step. Streptavidin horseradish peroxidase (HRP) was used, which reacts with an anti-human Annexin V antibody conjugated to biotin. It is possible to create a colored substance that is proportional to the amount of human Annexin V in the sample or standard. After adding acid to terminate the reaction, the Abs at 450 nm is measured using a microplate reader. For the standard curve preparation, seven human Annexin V standard dilutions and seven human Annexin V sample concentrations were calculated.

### Lactate Dehydrogenase Assay

The amount of LDH leaking out of EPS-treated cell lines was used to determine the plasma membrane integrity (Gurunathan et al., 2013). The cells were exposed to various EPSs at their IC_50_ concentrations for 24 h. LDH activity was determined in the culture after 3 h of incubation under standard conditions (K726-500) according to the manufacturer’s instructions (Biovision, Mountain View, CA, United States). The quantification of LDH is based on the color change caused by the reduction of NAD to NADH by 450 nm of LDH using a microplate reader. The activity of LDH was determined according to the following equations; LDH Activity level (nmol/ml) = **B**/(ΔT × **V**) × sample dilution; where ***B*** is the amount of NADH produced from ΔT (T2 - T1), **T1** is the time of first reading (A1) (in min), **T2** is the time of second reading (A2) (in min), and **V** is the volume of pretreated sample added into the reaction well (in ml).

### Characterization of Crude LAB-EPS_5_

Fourier transform infrared (FT-IR) spectroscopy was used to determine the major structural groups of the selected EPS_5_. The KBr technique was used to determine the EPS spectrum. Polysaccharide samples were crushed into KBr pellets at a sample ratio of 1:100. The infrared Fourier transform spectra were acquired using a Bruker Tensor 27 instrument with a resolution of 4 cm^–1^ in the range 4,000–400 cm^–1^ (Fontana et al., 2015). The monosaccharide content of EPS_5_ was determined using high-performance liquid chromatography (HPLC) as described by Yang et al. (2012). Briefly, the rehydrated EPS_1_ and EPS_5_ samples were derivatized with 1-phenyl-3-methyl-5-pyrazolone and analyzed by HPLC using a four-unit pump (Agilent Technologies, Wilmington, DE, United States) and a Shim-Pak VPODS column (4.6 × 150 mm) with Abs monitoring at 245 nm. An integrator was used to compare different sugar standards to the EPS sample’s retention time.

### Gene Expression Analysis

RNA was isolated from all cancer cell lines using a MagJET RNA purification kit, either untreated (negative control) or 24 h after treatment with EPS_5_ IC_50_. The quality and quantity of extracted RNAs were determined using 1% agarose gel electrophoresis and Nanodrop (Thermo Fisher Scientific, Inc., Wilmington, DE, United States). Quantitative real-time (qRT)-PCR was performed according to the manufacturer’s protocol using the Sybr Green Supermix RT-PCR kit, forward and reverse primers for each gene (Supplementary Table 1), and SYBR green PCR master mixture. The genes *BCL2*, *MCL1*, *Bax*, *Caspase 3*, *Caspase 8*, *Vimentin*, and *p53* were normalized with the housekeeping gene (GAPDH) and expressed about untreated cells. Following amplification, the obtained data were analyzed using the formula for relative gene expression (Winner et al., 1999; Livak and Schmittgen, 2001).

### Determination of the Proliferative Effect

Human periodontal ligament fibroblast cells (hPDLFCs) were extracted and grown according to Arslan and Kantarcioglu (2019). The Clinical Study Ethics Committee of Taif University accepted the research on an ethical basis. To examine the impact of various EPS_5_ concentrations on viability, hPDLFCs were plated of 1 × 10^4^ cells/well in 96-well plates, and viability was assessed using the MTT assay.

### Statistical Analysis

Minitab statistical software (17.1.0.0, Minitab Inc., Chicago, IL, United States) and GraphPad Prism version were used to interpret and process the data (9.0.0). To assess LDH activity and relative gene expression, the means of the groups were compared using an unpaired *t*-test. However, ANOVA single factor was used to analyze the synthesis of crude EPS by different probiotic bacterial strains and antioxidant activities of EPS. All measurements are presented as means ± SD. The one-way ANOVA test for variance was used to compare two or more groups, and the Tukey test was used for multiple comparisons. A *p*-value less than 0.05 was considered significant.

## Results

Table 1 depicts the production of EPSs by LAB and Bb, as well as their carbohydrate and protein contents. The amount of EPS produced by various bacterial strains varied significantly (*p* < 0.05). The EPS derived from bacteria ranged from 174.9 to 295.4 mg/l. *L. delbrueckii* ssp. *bulgaricus* DSM 20081^T^ produced the most EPS (EPS_5_, 295.4 ± 9.7), while *L. plantarum* DSM 20174 produced the least (EPS_9_, 174.9 ± 5.2). The carbohydrate and protein content of crude EPS varied with strain (*p* < 0.05). The total carbohydrate content ranged from 85.0 to 96.7%, while the protein content ranged from 3.3 to 9.3%. The average carbohydrate level for EPS_5_ is significantly higher (96.7 ± 0.3%) than for the other strains (*p* < 0.05). Furthermore, in the final lyophilized crude EPS samples, *S. thermophiles* DSM 24731-EPS_1_ had the lowest concentration of protein contamination.

**TABLE 1:** Production of crude EPS by different LAB and Bb strains.

EPS	EPS yield (mg/l)	Total carbohydrates content (%)	Protein content (%)
*Streptococcus thermophilus* DSM 24731-EPS_1_	240.0 ± 3.3^a^	93.0 ± 0.4^a^	3.3 ± 0.1^a^
*Lactobacillus lactis* ssp. *cremoris* DSM 20069^T^-EPS_2_	189.72 ± 3.4^b^	89.1 ± 0.08^b^	7.4 ± 0.2^b^
*Lacticaseibacillus casei* DSM 27537-EPS_3_	245.6 ± 3.2^a^	85.0 ± 0.1^c^	9.3 ± 0.2^c^
*Lactobacillus delbrueckii* ssp. *bulgaricus* DSM 20080-EPS_4_	222.4 ± 7.1^c^	93.1 ± 0.3^a^	8.3 ± 0.3^d^
*Lactobacillus delbrueckii* ssp. *bulgaricus* DSM 20081^T^-EPS_5_	295.4 ± 9.7^d^	96.7 ± 0.3^d^	4.3 ± 0.2^e^
*Limosilactobacillus fermentum* DSM 20049-EPS_6_	255.3 ± 4.3^e^	95.1 ± 0.1^e^	6.1 ± 0.2^f^
*Lactobacillus acidophilus* DSM 20079^T^-EPS_7_	213. 4 ± 4.2^c^	91.4 ± 0.4^f^	7.3 ± 0.1^b^
*Lacticaseibacillus rhamnosus DSM* 20021-EPS_8_	214.5 ± 4.1^c^	92.1 ± 0.4^f^	5.5 ± 0.2^g^
*Lactiplantibacillus plantarum* ssp. *plantarum* DSM 20174-EPS_9_	174.9 ± 5.2^f^	87.5 ± 0.2^g^	6.6 ± 0.1^h^
*Bifidobacterium longum* ssp. *longum* DSM 200707-EPS_10_	260. 3 ± 7.1^e^	94.1 ± 0.4^a^	5.7 ± 0.1^g^

*EPS, exopolysaccharide. Means in the same column followed by different letters are significantly different at p < 0.05.*

As shown in Tables 2, 3, the antioxidant capabilities of crude EPSs from 10 bacterial strains were evaluated *in vitro* by evaluating the activity of radical scavengers (DPPH and TAC, respectively). The DPPH free radical scavenging potential of various EPS concentrations increased significantly concentration-dependently (*p* < 0.05) (Table 2). At 20 mg/ml, the capacity of EPS to scavenge DPPH radicals ranged from 33.3% to 77.0%. *S. thermophiles* DSM 24731-EPS_1_ had the highest scavenging rate of all strains, at 77%, followed by *L. delbrueckii* ssp. *bulgaricus* DSM 20081^T^-EPS_5_, *B. longum* DSM 200707-EPS_10_, and *L. fermentum* DSM 20049-EPS_6_. *L. acidophilus* DSMZ 20079^T^-EPS_7_ had the least amount of scavenging activity (Table 2). The ability of a non-enzymatic antioxidant defense mechanism is represented by TAC. Table 3 shows that the antioxidant capacity ranged between 0.75 and 2.5%; the highest rate of inhibition was 2.5% for *L. delbrueckii* ssp. *bulgaricus* DSM 20081^T^-EPS_5_, followed by *S. thermophiles* DSM 24731-EPS_1_, *L. fermentum* DSM 20049-EPS_6_, and *B. longum* ssp. *longum* DSM 200707-EPS_10_, all of which had comparable degrading capabilities, and *L. acidophilus* DSM 20079 ^T^-EPS_7_ had the lowest rate of inhibition at 23.95%. Because EPS_1_, EPS_5_, EPS_6_, and EPS_10_ exhibited significantly greater antioxidant activity than other EPSs tested, they were chosen for subsequent assays in this study.

**TABLE 2:** Antioxidant activities (%) of EPS derived from LAB and Bb using DPPH radical scavenging assay.

EPS	DPPH scavenging rate (%) for different concentrations (mg/ml) of bacterial EPS					
	0.625	1.25	2.5	5.0	10.0	20
*S. thermophilus* DSM 24731-EPS_1_	0.00 ± 0.00^a^	14.3 ± 1.3^a^	31.3 ± 1.2^a^	41.8 ± 1.36^a^	56.4 ± 2.78^a^	77.0 ± 3.87^a^
*L. lactis* ssp. *cremoris* DSM 20069^T^-EPS_2_	0.00 ± 0.00^a^	10 ± 0.5^b^	18.0 ± 0.8b	24.4 ± 1.3^b^	27.6 ± 1.78^b^	34.5 ± 1.98^b^
*L. casei* DSM 27537-EPS_3_	10.05 ± 0.98^b^	11.04 ± 0.98^b^	12.0 ± 1.34^c^	19.0 ± 1.98^c^	25.5 ± 1.4^c^	35.5 ± 1.8^b^
*L. delbrueckii* ssp. *bulgaricus* DSM 20080-EPS_4_	17.10 ± 1.20^c^	33.2 ± 2.78^c^	37.8 ± 2.01^d^	48.5 ± 2.67^d^	50.4 ± 2.3^a^	56.3 ± 2.2^c^
*L. delbrueckii* ssp. *bulgaricus* DSM 20081^T^-EPS_5_	10.02 ± 2.13^b^	17.15 ± 2.67^a^	22.4 ± 1.43^e^	44.1 ± 2.54^ad^	65.0 ± 3.87^d^	75.2 ± 2.67^a^
*L. fermentum* DSM 20049-EPS_6_	16.04 ± 0.43^c^	17.06 ± 2.5^a^	18.0 ± 0.78^b^	29.1 ± 1.7^e^	55.1 ± 1.5^a^	60.2 ± 2.00^c^
*L. acidophilus* DSM 20079^T^-EPS_7_	14.03 ± 0.92^d^	17.08 ± 2.54^a^	23.0 ± 2.5^e^	30.0 ± 2.02^e^	33.0 ± 1.9^e^	33.3 ± 2.45^b^
*L. rhamnosus DSM* 20021-EPS_8_	10.02 ± 0.98^b^	11.01 ± 1.67^b^	12.0 ± 2.1^e^	19.1 ± 1.78^c^	25.5 ± 2.3^bc^	35.5 ± 2.02^b^
*L. plantarum* ssp. *plantarum* DSM 20174-EPS_9_	13.10 ± 0.78^d^	14.06 ± 2.99^a^	21.6 ± 2.0^e^	24.02 ± 2.88^b^	35.2 ± 2.65^e^	50.4 ± 2.78^d^
*B. longum* ssp. *longum* DSM 200707-EPS_10_	14.5 ± 2.24^cd^	23.04 ± 2.67^d^	31.4 ± 1.34^a^	42.0 ± 1.98^a^	54.0 ± 3.87^a^	65.3 ± 2.99^e^

*EPS, exopolysaccharide; DPPH, 2, 2 diphenyl-1-picrylhydrazyl. Each result is three times the mean ± SD. Means in the same column followed by different letters are significantly different at p < 0.05.*

**TABLE 3:** Antioxidant activities (%) of EPS derived from LAB and Bb using total antioxidant capacity (TAC) scavenging assay.

EPS	Total antioxidant capacity (%) for different concentrations (mg/ml) of bacterial EPS					
	0.625	1.25	2.5	5	10	20
*S. thermophilus* DSM 24731-EPS_1_	0.41 ± 0.1^a^	0.75 ± 0.3^a^	0.82 ± 0.5^a^	0.87 ± 0.1^a^	1.42 ± 0.1^a^	2.1 ± 0.2^a^
*L. lactis* ssp. *cremoris* DSM 20069^T^-EPS_2_	0.2 ± 0.1^b^	0.3 ± 0.1^b^	0.52 ± 0.1^a^	0.64 ± 0.1^b^	0.8 ± 0.1^b^	1.0 ± 0.1^b^
*L. casei* DSM 27537-EPS_3_	0.3 ± 0.2^ab^	0.42 ± 0.1^ab^	0.63 ± 0.2^a^	0.73 ± 0.1a^b^	0.85 ± 0.2^b^	1.3 ± 0.1^c^
*L. delbrueckii* ssp. *bulgaricus* DSM 20080-EPS_4_	0.37 ± 0.2^ab^	0.4 ± 0.3^ab^	0.55 ± 0.1^a^	0.65 ± 0.2a^b^	0.73 ± 0.3^b^	0.8 ± 0.1^b^
*L. delbrueckii* ssp. *bulgaricus* DSM 20081^T^-EPS_5_	1.21 ± 0.1^c^	1.47 ± 0.2^c^	1.64 ± 0.1^b^	1.75 ± 0.3^c^	1.8 ± 0.2^c^	2.5 ± 0.1^d^
*L. fermentum* DSM 20049-EPS_6_	1.1 ± 0.1^c^	1.3 ± 0.2^c^	1.5 ± 0.2^b^	1.62 ± 0.3^c^	1.73 ± 0.1^c^	1.87 ± 0.3^a^
*L. acidophilus* DSM 20079^T^-EPS_7_	0.23 ± 0.1^ab^	0.35 ± 0.1^ab^	0.45 ± 0.2^a^	0.5 ± 0.2^b^	0.64 ± 0.3^b^	0.75 ± 0.2^b^
*L. rhamnosus DSM* 20021-EPS_8_	0.1 ± 0.1^b^	0.21 ± 0.1^b^	0.47 ± 0.1^a^	0.6 ± 0.2^ab^	0.8 ± 0.3^b^	0.9 ± 0.2^b^
*L. plantarum* ssp. *plantarum* DSM 20174-EPS_9_	0.3 ± 0.2^ab^	0.4 ± 0.2^ab^	0.6 ± 0.1^a^	0.73 ± 0.1^ab^	0.9 ± 0.1^b^	1.23 ± 0.1^c^
*B. longum* ssp. *longum* DSM 200707-EPS_10_	0.5 ± 0.2^a^	0.8 ± 0.2^a^	1.1 ± 0.2^b^	1.4 ± 0.1^c^	1.55 ± 0.3^ac^	1.73 ± 0.2^a^

*EPS, exopolysaccharide. Means in the same column followed by different letters are significantly different at p < 0.05.*

**FIGURE 2:**
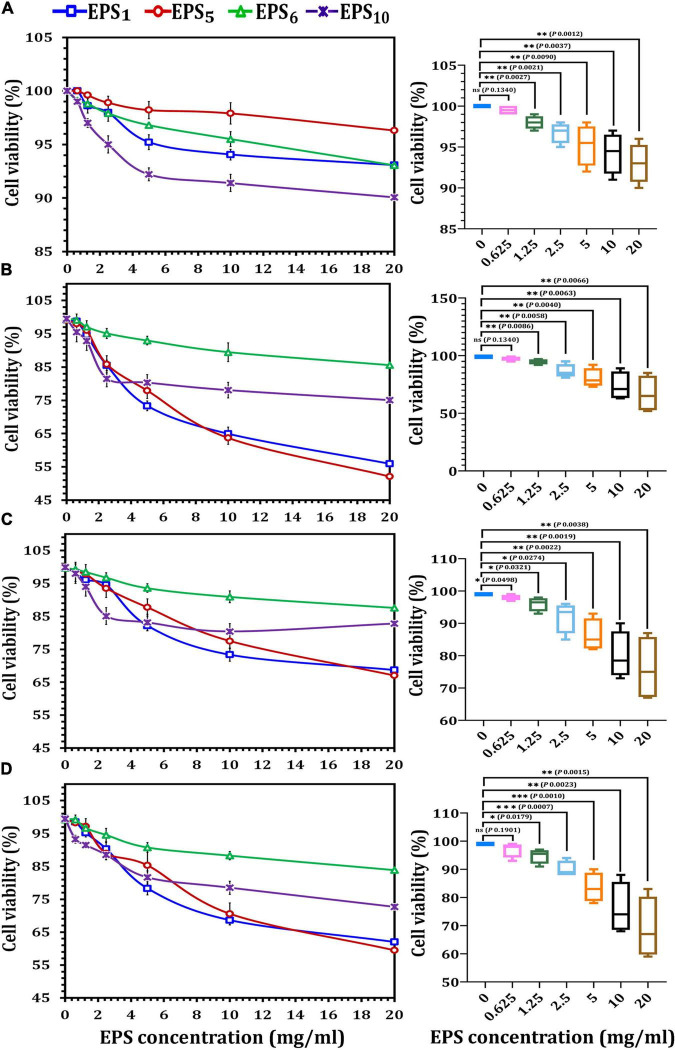
Cytotoxicity assessments of exopolysaccharides on different cancer cell lines using MTT assay. **(A)** On HEK293 cell line, **(B)** on MCF7 cell line, **(C)** on CaCo2 cell line, and **(D)** on HepG2 cell line. EPS_1_, EPS derived from *S. thermophilus* DSM 24731; EPS_5_, EPS derived from *L. delbrueckii* ssp. *bulgaricus* DSMZ 20081^T^; EPS_6_, EPS extracted from *L. fermentum* DSMZ 20049, and EPS_10_, EPS derived from *B. longum* ssp. *longum* DSMZ 200707.

As illustrated in Figure 2, MCF7, CaCo2, and HepG2 cells all responded differently to the MTT cytotoxicity experiment. All of the EPSs inhibited cells in a concentration-dependent manner, with varying effects on various cell lines. After 24 h of treatment with the highest doses (20 mg/ml) of EPS_1_, EPS_5_, EPS_6_, and EPS_10_, the viability percentages for HEK 293 cells were 93.07, 96.3, 93.05, and 90.07 mg/ml, respectively (Figure 2A); MCF7 cells were 55.9, 52.1, 85.52, and 75.01 mg/ml, respectively (Figure 2B); CaCo2 cells were 68.69, 67.0, and 87.59 mg/ml, respectively (Figure 2C) and HepG2 cells were 62.0, 59.5, 83.86, and 72.66 mg/ml, respectively (Figure 2D). As indicated in Figure 2A, the impact of the chosen EPSs on viability (%) of HEK 293 cells showed that at 0.625 mg/ml (98.9%), the viability rate of HEK 293 cells was not significantly different (*p* = 0.1340). When the growth rate of HEK 293 cells was compared to that of cells treated with EPS at doses of 0.125 mg/ml (*p* = 0.0027), 2.5 mg/ml (*p* = 0.0021), 5 mg/ml (*p* = 0.0090), 10 mg/ml (*p* = 0.0037), and 20 mg/ml (*p* = 0.0012), a significant difference was observed. At 20 mg/ml, MCF7 viability decreased by more than 30% as compared to control viability, a significant difference of *p* = 0.0066. On the other hand, at EPSs concentrations of 0.625 (*p* = 0.1340), the MCF7 viability rate was not significantly different (Figure 2B). A significant difference was detected when the CaCo2 viability% of the control was compared to cells treated with EPSs concentrations of 5 mg/ml (*p* = 0.0022), 10 mg/ml (*p* = 0.0019), and 20 mg/ml (*p* = 0.0038) (Figure 2C). When the HepG2 viability% of the control was compared to cells treated with EPSs doses of 2.5 mg/ml (*p* = 0.0007) and 5 mg/ml (*p* = 0.0010), a very significant difference was seen (Figure 2D). Generally, according to the obtained data for EPS_1_ and EPS_5_, the extract’s potency was in the order MCF7 > HepG2 > CaCo2 at various concentrations; however, EPS_6_ and EPS_10_ had a strong inhibitory rate in the order HepG2 > MCF7 > CaCo2. Furthermore, EPSs were found to have a minimal cytotoxic effect on HEK 293 cells. According to the findings, EPS greatly reduced the viability of different cancer cells while having no impact on normal cells.

**FIGURE 3:**
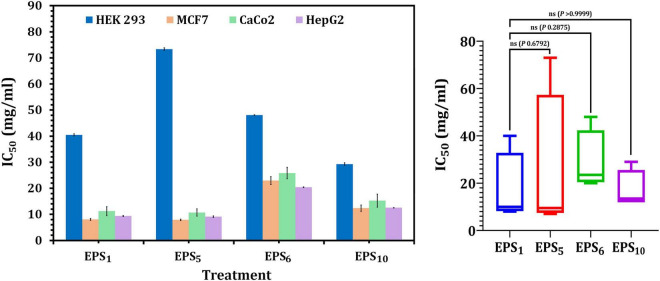
Cytotoxicity of EPSs against HEK293, MCF7, CaCo2, and HepG2 using MTT assay. EPS_1_, EPS derived from *S. thermophilus* DSM 24731; EPS_5_, EPS derived from *L. delbrueckii* ssp. *bulgaricus* DSMZ 20081^T^; EPS_6_, EPS extracted from *L. fermentum* DSMZ 20049, and EPS_10_, EPS derived from *B. longum* ssp. *longum* DSMZ 200707.

As shown in Figure 3, the IC_50_ values of EPS_1_, EPS_5_, EPS_6_, and EPS_10_, for MCF7 cells were 8.06, 7.91, 22.97, and 12.36 mg/ml, respectively; for CaCo2, they were 10.69, 11.22, 25.85, and 15.23 mg/ml, respectively; and for HepG2 cells, they were 9.12, 9.37, 20.40, and 12.54 mg/ml, respectively. EPS_5_ had the most active components and exhibited the highest cytotoxicity with the lowest IC_50_ values on MCF7, CaCo2, and HepG2 (7.91, 10.69, and 9.12 mg/ml, respectively). EPS_6_, on the other hand, had the highest IC_50_ values against MCF7, CaCo2, and HepG2 at 12.36, 15.23, and 12.54 mg/ml, respectively. It is worth noting that untreated control cells retained a high viability. While EPS decreased the viability of normal HEK 293, their IC_50_ values of 29.26–85.7 mg/ml were significantly greater than those of cancerous cells (Figure 3). These results demonstrated that different EPSs may have a significant influence on EPS biological activities and that the inhibitory feature differed with human cell strains and produced severe cytotoxicity at large doses.

**FIGURE 4:**
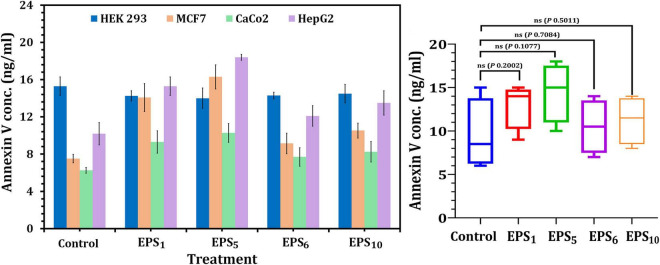
Apoptosis assessment using annexin V ELISA kit on cancer cell lines. EPS_1_, EPS derived from *S. thermophilus* DSM 24731; EPS_5_, EPS derived from *L. delbrueckii* ssp. *bulgaricus* DSMZ 20081^T^; EPS_6_, EPS extracted from *L. fermentum* DSMZ 20049, and EPS_10_, EPS derived from *B. longum* ssp. *longum* DSMZ 200707.

Annexin V has been shown to attach to apoptotic phosphatidylserine-releasing cells and decrease their pro-coagulant and pro-inflammatory activity. The obtained results showed that cellular apoptosis was triggered in MCF7, CaCo2, and HepG2-treated cells following the IC_50_ value treatment of the selected crude EPSs, as shown in Figure 4. In MCF7, CaCo2, and HepG2 cells treated with EPS_5_-IC_50_ values, the greatest Annexin V quantities (18.4, 10.28, and 16.30 ng/ml, respectively) were observed in comparison to other EPS treatments. On the other hand, apoptosis assays after EPS_6_ treatment revealed that total endogenous Annexin V levels were low in a variety of cancer cells, suggesting that EPS_6_ is an ineffective anticancer agent. Generally, the total endogenous Annexin V levels in cells treated with various EPSs indicated that the concentrations of Annexin V were not significantly different (*p* = 0.2002, 0.1077, 0.7084, and 0.5011) in cells treated with EPS_1_, EPS_5_, EPS_6_, and EPS_10_ compared to control cells (untreated) (Figure 4).

**FIGURE 5:**
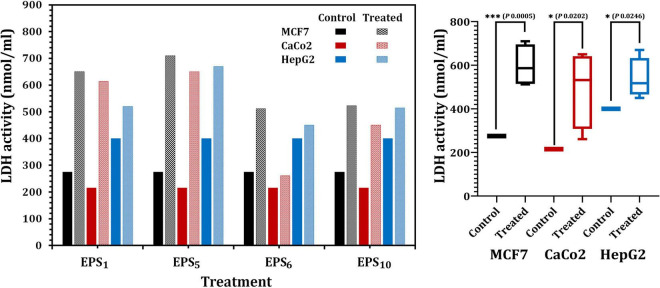
Lactate dehydrogenase (LDH) activity in MCF7, CaCo2, and HepG2 treated with different EPSs. EPS_1_, EPS derived from *S. thermophilus* DSM 24731; EPS_5_, EPS derived from *L. delbrueckii* ssp. *bulgaricus* DSMZ 20081^T^; EPS_6_, EPS extracted from *L. fermentum* DSMZ 20049, and EPS_10_, EPS derived from *B. longum* ssp. *longum* DSMZ 200707.

The quantity of LDH released, a soluble cytoplasmic enzyme, was utilized to determine the degree of degradation and leakage in cell membranes impacted by the IC_50_ of EPS_1_, EPS_5_, EPS_6_, and EPS_10_ (Figure 5). Regarding MCF7, LDH activity in EPS_1_ and EPS_5_-treated cells significantly increased by 2.36 and 2.58 times, respectively, related to the control (untreated) MCF7 cells (*p* < 0.0001) as illustrated in Figure 5. Furthermore, in the case of CaCo2, LDH activity increased by 2.85- and 3.02-fold, respectively, in the cells treated with IC_50_ values of each EPS_1_ and EPS_5_ relative to the untreated CaCo2 cells. LDH activity was highest (670 nmol/ml) in HepG2 cells treated with EPS_5_. As illustrated in Figure 5, EPS_5_ had a considerably higher LDH activity than EPS_1_ (*p* = 0.0001), followed by EPS_10_ and EPS_6_. In comparison to the control cell, LDH leakage concentrations in MCF7 cells treated with various EPSs varied significantly (*p* = 0.0202). In general, EPS_5_ demonstrated significant antitumor activity in the three types of cell lines analyzed in our research, based on the data obtained on the antitumor abilities of the tested EPSs. As a result, EPS_5_ will be further characterized and used in the following biological applications.

**FIGURE 6:**
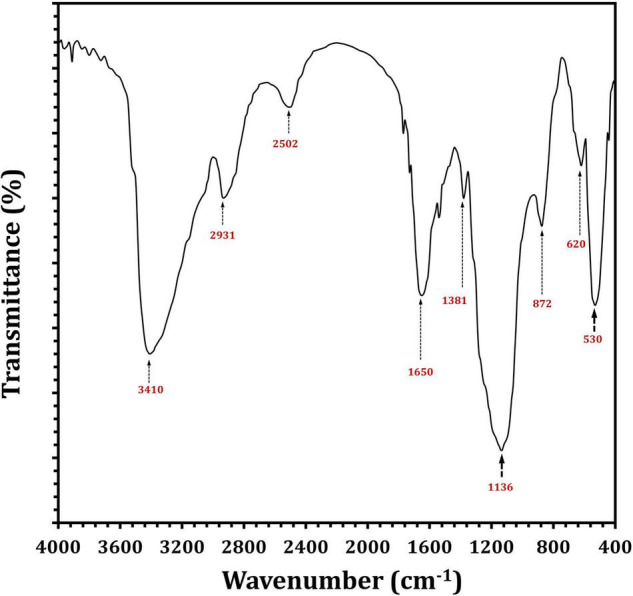
FTIR spectra of *L. delbrueckii* ssp. *bulgaricus* DSM 20081^T^-derived EPS_5_.

Fourier transform infrared analysis showed a variation in the functional groups formed in EPS_5_, as shown in Figure 6. There were eight absorbance bands in the FT-IR spectrum of EPS_5_; 3410, 2931, 2502, 1650, 1381, 1136, 872, 620, and 530 cm^–1^. Table 4 displays the retention period, area under the curve, and monosaccharide composition of EPS_5_ samples as measured by HPLC. The EPS samples’ sugar content varied substantially (*p* < 0.05). EPS_5_ included a high concentration of sugars such as rhamnose, mannose, ribose, glucose, and galactose. The sugar concentrations in EPS_5_ varied from 1.7938 to 38.8584 mg/100 g, with galactose being the greatest concentration. The ratio of ribose, rhamnose, glucose, mannose, and galactose in EPS_5_ was 1:2.7:7.4:5.1:12.5. Consequently, the EPS_5_ samples were heteropolysaccharides based on their sugar content.

**TABLE 4:** Determination of monosaccharide composition of *L. delbrueckii* ssp. *bulgaricus* DSM 20081^T^-EPS_5_ using HPLC.

Monosaccharides	EPS_5_	
	RT	Area under curve
Ribose	8.788	1.6
Rhamnose	13.66	1.06
Glucose	16.067	1.34
Mannose	17.092	4.81
Galactose	18.058	6.52

*RT, retention time.*

**FIGURE 7:**
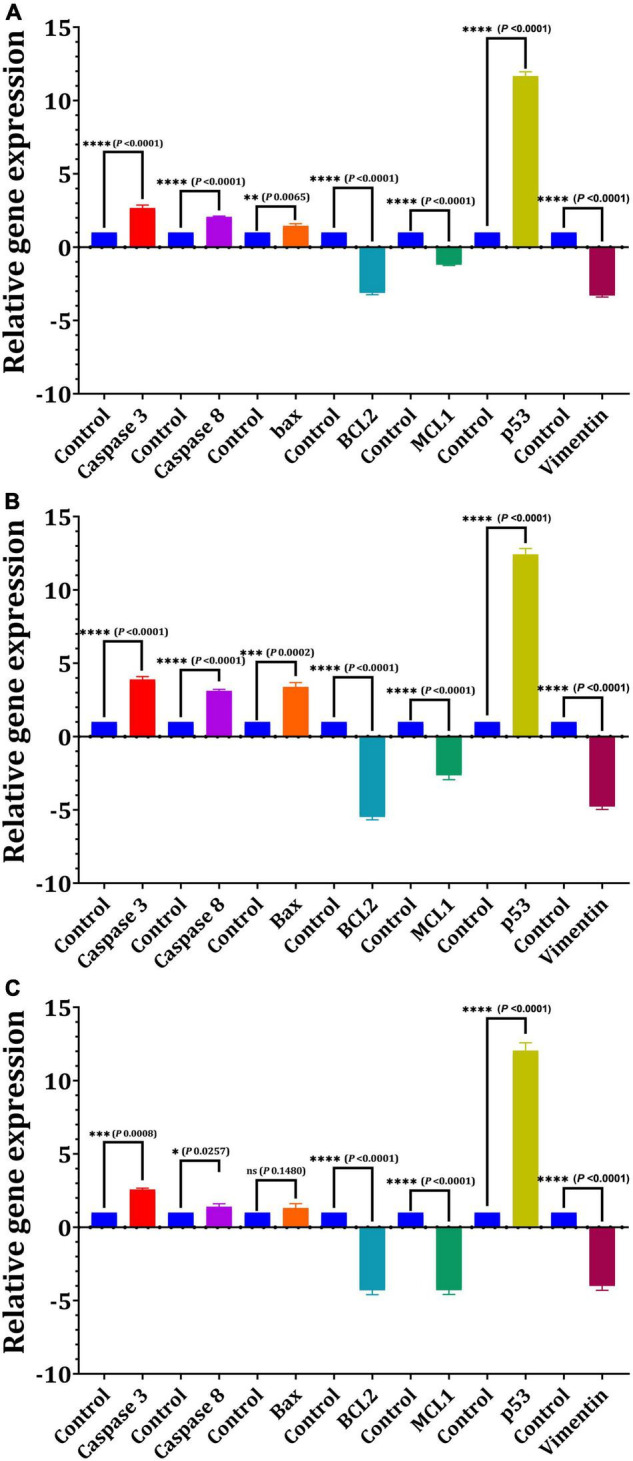
Effect of *L. delbrueckii* subsp. *bulgaricus* DSM 20081^T^-EPS_5_ on the relative expression of genes linked to apoptosis in different cell lines. **(A)** MCF7, **(B)** CaCo2, **(C)** HepG2.

For 24 h, we used quantitative real-time PCR to determine the mRNA levels of apoptotic and cell cycle-related genes in response to the IC_50_-EPS_5_ value (Figure 7). The data of gene expression revealed that EPS_5_ with IC_50_ value was significantly upregulated *Bax* gene expression in CaCo2 cells (*p* = 0.0065) compared with that in control (Figure 7A). However, a marked reduction in expression of the *BCL2* and *MCL1* (*p* = 0.0001) genes were observed in treated CaCo2 cells (Figure 7A). Additionally, the results indicated that EPS_5_ administration increased the expression of apoptotic genes *Caspase 3* and *Caspase 8*. The most significant upregulation of the *p53* gene was detected in CaCo2 cells treated with IC_50_-EPS_5_ (*p* = 0.0001). In comparison, the least significant upregulation of the *Bax* gene was found in MCF7 cells treated with the same concentration of EPS_5_ (*p* = 0.0002) (Figure 7B). Additionally, in the treated MCF7 cells, *BCl2*, *MCL1*, and *Vimentin* genes observed a downregulation at their expression level (*p* < 0.0001). However, the *Caspase 3*, *Caspase 8*, and *Bax* genes recorded upregulation of expression (Figure 7B). As demonstrated in Figure 7C, *BCL2* expression was strongly downregulated in treated HepG2 cells, as has been the case with *MCL1* and *Vimentin* expression, dramatically lowering the levels of the studied genes relative to control (*p* = 0.0001). EPS_5_, on the other hand, resulted in a significant increase in the expression of p53 (12.04) as compared to the control (*p* = 0.0001) but had no influence on *Bax* expression.

**FIGURE 8:**
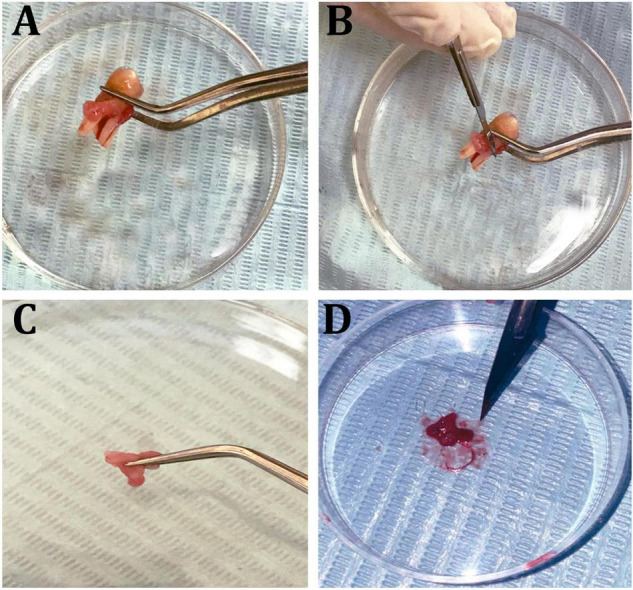
Isolation steps of human periodontal ligament fibroblast cells (hPDLF). **(A)** Healthy molar tooth, **(B)** scraping periodontal layers, **(C,D)** mincing of the periodontal membrane.

**FIGURE 9:**
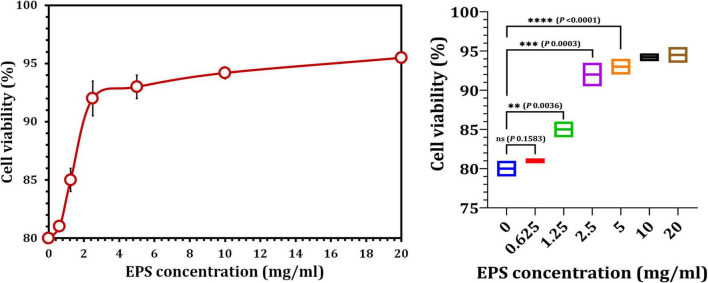
The effect of crude EPS_5_ derived from *L. delbrueckii* ssp. *bulgaricus* DSM 20081^T^ on cell viability in hPDLF cells using MTT assay.

Cultured hPDLFCs were successfully extracted from healthy molar teeth (Figure 8). After 24 h of co-incubation with various concentrations of crude EPS_5_, cell viability was determined using the MTT assay. As illustrated in Figure 9, the viability of hPDLFCs was significantly increased following exposure to crude EPS. When hPDLFCs were treated with EPSs at a concentration of 0.625 mg/ml, the viability rate was not significantly different from that of untreated cells (*p* = 0.1583). When the viability percent of untreated hPDLFCs was compared to cells treated with 2.5 mg/ml (*p* = 0.0003) and 5 mg/ml (*p* = 0.0003) EPSs, a significant difference (*p* = 0.0001) was observed (Figure 9).

## Discussion

In this study, LAB (*n* = 9) and Bb (*n* = 1) were used to extract EPS, and their EPS yield was determined. The EPS obtained demonstrated a high degree of concentration- and strain-dependent variation (*p* < 0.05). When compared to other tested strains, *L. delbrueckii* ssp. *bulgaricus* DSM 20081^T^ produced the most EPS (295.49 ± 0.7). Tukenmez et al. (2019) reported that *L. delbrueckii* ssp. *bulgaricus* B3 produced the highest amount of EPS (449.0 ± 0.4 mg/l). According to Sungur et al. (2017), two strains of *L. gasseri* produced 242 and 255 mg/l of EPS. *Bifidobacterium bifidum* WBIN03 produced EPS at a concentration of 241.20 mg/l, whereas *L. plantarum* R315 produced EPS at a concentration of 290.17 mg/l (Li S. et al., 2014).

Reactive oxygen species (ROS) and oxygen-centered free radicals are byproducts of a number of metabolic and physiological processes in the body (Thibessard et al., 2004). Increased levels of ROS degrade proteins, DNA, and lipids, causing additional tissue damage and organ dysfunction. This causes serious side effects in humans, such as carcinoma, arteriosclerosis, osteoarthritis, and neurodegeneration (Li C. et al., 2014). Antioxidants are substances that prevent or postpone oxidation. Tumor cells produce ROS as a result of increased metabolic activity and a variety of other tumorigenic processes, which promotes tumor cell proliferation and survival, mesenchymal migration and penetration, genetic mutations, and angiogenesis. Antioxidant-based therapy that reduces ROS generation may be used to delay or even prevent tumor development (Liou and Storz, 2010; Gurunathan et al., 2013). Numerous research have been conducted on EPS derived from probiotic bacteria, which may possess antioxidant activities (Adebayo-Tayo and Fashogbon, 2020; Wu et al., 2021). According to our findings, the radical scavenging capacity of EPS_1_ (77%) and EPS_5_ (75.2%) significantly contributes to antioxidant activity. Adebayo-Tayo and Fashogbon (2020) evaluate the antioxidant activity of EPS produced by wild type EPSWLD *L. delbrueckii* ssp. *bulgaricus* and mutant EPSMLD *L. delbrueckii* ssp. *bulgaricus* using the DPPH system and other biological tests. The antioxidant potential of EPSWLD and EPSMLD was found to be between 1.21 and 1.80% and between 0.41 and 1.42% for their DPPH-scavenging capacities (37.5–73.4% *vs*. 37.5–65.6%, respectively). El-Adawi et al. (2012) reported that extracellular extracts of *B. longum* and *L. plantarum* had a DPPH scavenging capacity of 89.8 and 89.8%, respectively. The entire culture of *L. delbrueckii* ssp. *bulgaricus* DSM 20081^T^ possessed the highest DPPH radical scavenging activity. According to Li S. et al. (2014), *B. bifidum* WBIN03 (B-EPS) and *L. plantarum* R315 (L-EPS) both had a high DPPH scavenging potential that increased with EPS concentration. Clearly, 70 mg/ml EPSs had a high DPPH scavenging potential when they compared the DPPH scavenging behavior of different concentrations of B-EPS (30 mg/ml, 94.4%) and L-EPS (70 mg/ml, 94.49%). Recent research has revealed that the antioxidant ability of EPS is affected by a number of factors, including molecular weight, monosaccharide composition, monosaccharide molar ratio, and glycoside bonding (Wu et al., 2021). As a result, the significant antioxidant activity of EPS could be attributed to its chemical groups, which include the hydroxyl group, carbon-free radicals, and the sulfated group (Wang et al., 2015).

Cancer is a disease that causes uncontrollable cell proliferation, resulting in organ damage and, eventually, death. The death toll from various types of cancer is steadily rising year after year. Current chemo and radiation therapy for cancer patients harms both tumor and normal cells (El Ghany et al., 2015; Zhang et al., 2017). The current research aims to produce antitumor agents that have fewer side effects than currently available synthetic pharmaceuticals. As a result, the antitumor properties of EPS are being investigated as a potential cancer treatment in this study. The cytotoxicity of EPSs against human breast, colon, and liver cancer cells was determined, as well as their role in apoptotic gene expression. The majority of anti-cancer medications have been shown to be cytotoxic to healthy cells, highlighting the critical need for safe alternatives in biomedicine (Zhang et al., 2017). Mohanta et al. (2017) demonstrated that the MTT test is one of the most advanced methods for determining the cytotoxic effects of cancerous cells. It is based on the reduction of MTT into formazan by mitochondrial succinate dehydrogenase in the mitochondria of metabolically active cells. The results of the cytotoxicity tests in this experiment revealed that treatment with EPSs elicited different cellular responses depending on the cause of cell death and the sensitivity of the treatment. Specifically, the IC_50_ values for EPSs on normal mammalian cells (HEK 293) ranged from 85.7 to 29.68 mg/ml. Nami et al. (2015) found that *Enterococcus lactis* IW5 secretions had no negative effects on normal cells, with 95% of the cells developing normally. Furthermore, a dosage of 2–5 mg/ml of new purified EPS *L. acidophilus* 20079 in healthy mammalian cells was shown to have a selectivity index of 1.96–51.3 for destroying cancerous cells (El-Deeb et al., 2018).

Exopolysaccharides from *L. plantarum* has not been found to be harmful in normal fibroblast cells L929 until a concentration of 50 mg/ml is reached (Ismail and Nampoothiri, 2013). The viability percentage of L929 is greater than 60 after treatment with 40 mg/ml EPS from *L. paracasei* and *Lactobacillus brevis* on L929 and HT29. On the other hand, HT29 has a survival rate of less than 20% (Mojibi et al., 2019). Based on the multiple cancerous cell death pathways and related sensitivity to treatment, we can assume that cytotoxicity experiments revealed the presence of non-identical cellular responses to EPS treatment. EPS_5_ had the most active components and exhibited the lowest IC_50_ values on MCF7, CaCo2, and HepG2 (7.91, 10.69, and 9.12 mg/ml, respectively, *p* < 0.05). Vidhyalakshmi and Vallinachiyar (2013) demonstrated that EPS derived from *Bacillus* sp. has a high potential for cytotoxicity against MCF7 cells at low concentrations while having no effect on normal cells. Additionally, Liu et al. (2011) found that *L. casei* 01 EPS inhibited HT29 growth at doses ranging from 5 to 100 μg/ml. *L. fermentum* extracts inhibited colon cancer cell growth while enhancing non-cancerous colon cell growth (Kahouli et al., 2015). Their findings on cancer cells suggest that the presence of propionate and butyrate may contribute to the probiotic bacteria’s selectivity, as histone deacetylase inhibitors (HDACs) stimulate proliferation in healthy colon cells but kill cancer cells (Kahouli et al., 2015). Nami et al. (2015) reported that the *E. lactis* IW5 byproducts reduced the viability of several types of cancer cells, including HeLa, MCF7, AGS, and HT-29, and that the primary mechanism underlying this effect was the activation of apoptosis in cancer cells.

Annexin V analysis revealed a decrease in the percentage of viable cells in all investigated cell lines with increased EPSs concentrations. A rise in the concentration of Annexin V in treated cells indicates that EPS induces apoptosis. The investigation revealed that treatment with the IC_50_ of EPS_5_ resulted in a significantly increased number of apoptotic cells (18.4, 10.28, and 16.3 ng/ml) on MCF7, CaCo2, and HepG2, respectively, followed by treatment with EPS_1_, whereas treatment with the IC_50_ of EPS_6_ resulted in a significantly decreased number of apoptotic cells. Apoptosis is primarily defined by changes in the morphology and biochemistry of the cells. EPS of *L. paracasei* and *L. brevis* inhibited the proliferation of HT29 cancer cells (Mojibi et al., 2019). When anti-proliferative activity and cell growth are compared 24 h after treatment with *L. paracasei* or *L. brevis* EPS at a concentration of 40 mg/ml, anti-proliferative activity and cell death increase from 36 to 80% and from 40 to 90%, respectively. *S. thermophilus* CH9-EPS-3a demonstrated greater anticancer activity *in vitro* against human liver cancer HepG2 cells, and the antitumor efficacy of EPS’s-3a was associated with cell apoptosis in HepG2 cells (Liu et al., 2019). Normal cells possess significantly fewer anti-proliferative and inhibitory properties than cancerous cells. Increased LDH leakage into the culture supernatant is also indicative of EPS-induced membrane damage (Deepak et al., 2016). LDH activity was found to be significantly greater in cancer cells treated with EPS_5_ than in cancer cells treated with EPS_1_ (*p* = 0.0001), followed by EPS_10_ and EPS_6_, confirming disruption of the plasma membrane and subsequent LDH dispersion into extracellular space (Fotakis and Timbrell, 2006).

It has been demonstrated that EPSs perform a variety of bioactive functions, and as a result, these functions are typically influenced positively by the EPS’s physicochemical properties (Wang et al., 2014; Li et al., 2016). This indicates a distinction between the functional groups defined in EPS_5_ when discussing FT-IR results. The strongest bands in the 3,410 cm^–1^ region are indicative of the presence of the EPS_5_ hydroxyl-stretching vibration (Abdhul et al., 2014). The bands at 2,931 cm^–1^ are produced by CH_2_ stretching vibrations, whereas the bands at 1,650 cm^–1^ are produced by –OH bending vibrations. Absorption at 1,381 cm^–1^ may be due to symmetric CH_3_ bending. At 1,136 cm^–1^, vibrations stretching the glycosidic bond (C–O–C) were responsible for the strong absorption (Adebayo-Tayo and Fashogbon, 2020). Additionally, strong infrared absorptions at 872 cm^–1^ indicated that EPS_5_ was β-anomeric (Pan and Mei, 2010). The monosaccharide of EPS_5_ was investigated using HPLC. The EPS_5_ is primarily composed of various sugars with molecular ratios 1:2.7:7.4:5.1:12.5, reflecting ribose:rhamnose:glucose:mannose:galactose, respectively.

The most critical biochemical process that occurs during early apoptosis is phosphatidylserine translocation. Several biochemical pathways converge during apoptosis, resulting in the activation of a family of cysteine-dependent aspartate-directed proteases (caspases). Although caspase-dependent or caspase-independent mechanisms can regulate apoptosis, the latter is more prevalent because the majority of cells initiate apoptosis *via* caspase activation (Gamal-Eldeen et al., 2009). We investigated the effect of *L. delbrueckii* ssp. *bulgaricus* DSMZ 20081^T^-EPS_5_ on apoptotic marker gene expression in MCF7, CaCo2, and HepG2 cells as a result of the antitumor data obtained. The relative expression of the apoptotic genes *Bax*, *BCL2*, *Caspase 3*, *Caspase 8*, and *MCL1* was determined in this experiment. Board proteins, such as *BCL2*, regulate both cell damage and proliferation. *BCL2* family proteins are required for mitochondria-mediated apoptosis, as they maintain the mitochondrial membrane’s integrity (Aouacheria et al., 2017).

The mechanism by which polysaccharides induce apoptosis in cancerous cells was investigated using BCL2 family proteins such as *BCL2*, which inhibits apoptosis, and *Bax*, which induces apoptosis. The results of the gene expression analysis indicated that EPS_5_ increased *Bax* gene expression in the cell lines examined. However, cells treated with EPS_5_ showed a significant decrease in *BCL2* gene expression. Numerous studies have shown that tumor cells treated with LAB EPS expressed significantly less *BCL2*. Tukenmez et al. (2019) found that *L. delbrueckii* ssp. *bulgaricus* B3-EPS inhibited time-dependent proliferation and induced apoptosis by increasing *Bax*, *Caspase 3*, and *Caspase 9* expression and decreasing *BCL2* and *Survivin* expression. Cell-bound exopolysaccharides (cb-EPS) derived from *L. acidophilus* 606 inhibited the growth of colon cancer cells HT-29 by activating the *Bax* gene (Yang et al., 2007). Additionally, the *MCL1* gene, a member of the *BCL2* family of anti-apoptotic proteins that promotes apoptosis activation *via* mitochondrial pathways in C32 melanoma cells, inhibits apoptosis in a variety of cancers *Bak* and *Bax* pro-apoptotic proteins (Beberok et al., 2020). Caspases are involved in the regulation of the majority of molecules involved in cell death. Caspases involved in apoptosis can be classified as initiators (*Caspases 2*, *8*, *9*, and *10*) or effectors (*Caspases 3*, *6*, and *7*) (Wang et al., 2016).

Given the critical role of *Caspase 3* in apoptosis and the fact that many well-characterized breast cancer cell lines have altered *Caspase 3* expression, RT-PCR was performed using *Caspase 3*-specific primers in treated MCF7 cells. *Caspase 3* was significantly overexpressed in treated MCF7 cells compared to other cancer cells tested, according to our findings. *Caspase 3* activity was detected in response to a variety of apoptotic factors, including chemotherapeutic agents, radiotherapy, and cytokines. Selective *Caspase 3* inhibition, on the other hand, is associated with cell death inhibition. It has been postulated that a lack of *Caspase 3* expression allows apoptosis-resistant breast cancer cells to respond to apoptotic stimuli like chemotherapy and radiotherapy. Several findings have significant clinical implications for using *Caspase 3* as a disease marker for breast cancer as well as a potential therapeutic target (Devarajan et al., 2002). Northern blot analysis was used to confirm the absence of *Caspase 3* expression in breast cancer cells. *Caspase 3* mRNA levels in breast cancer cells were 10- to 50-fold lower than in normal breast tissue. In drug-sensitive MCF7 cells, *Caspase 3* mRNA was truncated by a 125-bp transcript deletion. Additionally, it was established that drug-resistant (MCF7/DOX) MCF7 cells derived from continuous MCF7 cell culture had a maximum *Caspase 3* transcript duration in the presence of doxorubicin (Devarajan et al., 2002). The significant increases in *Caspase 8* and *Caspase 3* genes in treated cells in our results are consistent with Chang et al. (2013). *Caspase 3* was expected to be an adequate death protease catalyzing protein breakdown. *Caspase 8* is found at the apex of an apoptotic cascade, where it causes proteolytic activation of members of the downstream caspase family, which contributes to apoptosis (Chang et al., 2013). Two pathways triggered the initiator: the first is regulated by *Caspase 8*. It includes the insertion of cell death ligands, which then activates *Caspase 8* and *Caspase 3*, and the later involves apoptosis mediated by *Caspase 9* mitochondria (Karpel-Massler et al., 2017).

The *p53* tumor suppressor can also regulate pro-apoptotic genes related to both intrinsic and extrinsic pathways (Chipuk et al., 2003). El-Deeb et al. (2018) observed that after treating CaCo2 cells with *L. acidophilus* LA-EPS-20079 EPS, *p53* gene expressions increased. EPS_5_ increased the level of *p53* transcript in the tested CaCo2 cell line, implying that a *p53*-dependent pathway is active in the apoptotic mechanism of CaCo2 cells. To maintain cellular integrity and tolerance to stress, normal mesenchymal cells produce *Vimentin*, a protein belonging to the intermediate filament (IF) family (Chipuk et al., 2003). *Vimentin* aberrant overexpression has been linked to a higher level of aggressiveness in cancer cells, including melanoma, breast cancer, gastrointestinal cancers, and nervous system tumors. *Vimentin* overexpression in cancer is linked to increased tumor development, invasion, and a bad prognosis; nevertheless, the function of *Vimentin* in the development of cancer is still unknown. To our knowledge, no research on the effect of EPS on *Vimentin* gene expression regulation in MCF7 has been conducted. McInroy and Määttä (2007) demonstrated that silencing *Vimentin* expression inhibits the migration and invasion of colon and breast cancer cell lines. They demonstrated that downregulation of *Vimentin* expression impairs migration in both scratch wound experiments and invasion studies using collagen-coated cell culture inserts.

**FIGURE 10:**
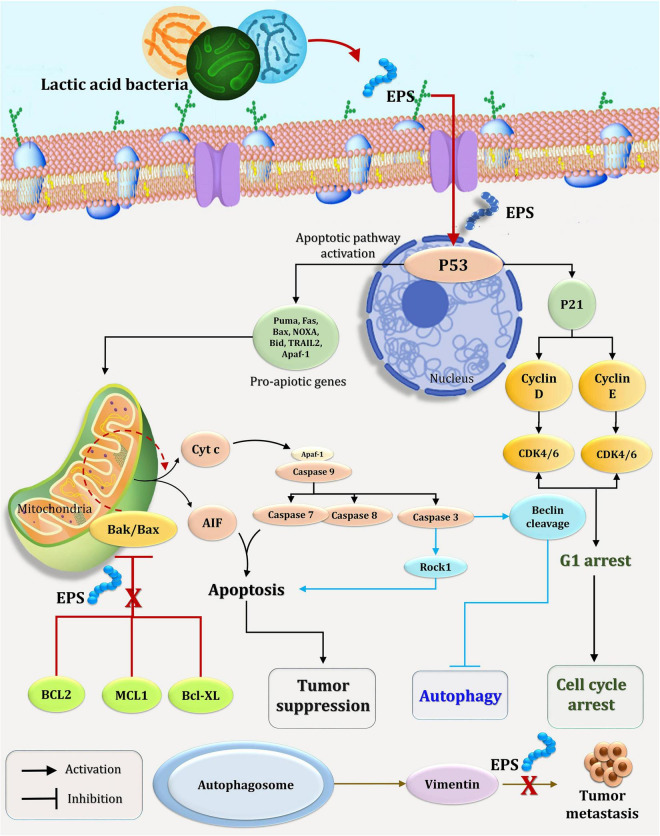
A possible mechanism for EPS_5_’s effect on the cell cycle and apoptosis in cancer cells.

Recent research has established the efficacy of polysaccharides in inducing apoptosis in cancer cells by focusing on signaling molecules (Alwarsamy et al., 2016). As illustrated in Figure 10, polysaccharides exert their antitumor activity *via* two distinct mechanisms: the *BCL2*-regulated (also known as intrinsic, mitochondrial, or stress) pathway, which is activated by cytokine deprivation, ER stress, or DNA damage, and the death receptor (also known as extrinsic) pathway, which is activated by ligation of members of the tumor necrosis factor receptor (TNFR) family bearing an intracellular death domain (Green, 2005; Czabotar et al., 2014; Zhang et al., 2017). Overexpression of the pro-apoptotic *BH3*-only members (*BIM*, *PUMA*, *BID*, *BMF*, *BAD*, *BIK*, *NOXA*, and *HRK*) of the *BCL2* protein family, which are members of the pro-apoptotic *BH3*-only subfamily, promotes cell death through the *BCL2*-regulated apoptotic pathway. The *BH3*-only proteins interact with and block pro-survival *BCL2* proteins (*BCL2*, *BCLXL*, *MCL1*, *BCLW*, and *A1/BFL1*), hence activating the cell death effectors *BAX* and *BAK* (the pro-apoptotic multi-BH domain members of the *BCL2* family that may also contain *BOK*) (Ke et al., 2015; Llambi et al., 2016). Certain *BH3*-only proteins have been shown to directly activate *BAX/BAK* (Green, 2005; Youle and Strasser, 2008). When *BAX*/*BAK* is activated, the mitochondrial outer membrane (MOMP) is permeabilized, triggering the activation of a cascade of aspartate-specific cysteine proteases (Zhang et al., 2007) and its activator APAF-1 (Cecconi et al., 1998) that dismantle cells (Figure 10). In comparison, the downregulation system induces apoptosis by binding and activating *Procaspase-8* at ligated death receptors in the plasma membrane *via* the adaptors FADD and, in rare cases, TRADD. In so-called type 1 cells, activation of *Caspase 8* followed by activation of effector caspases (*Caspases 3* and *7*) is sufficient to successfully induce apoptosis (e.g., thymocytes). On the other hand, effective cell death in so-called type 2 cells (e.g., hepatocytes) requires caspase cascade amplification *via* crossover activation of the *BCL2*-regulated apoptotic pathway, which is accomplished through *Caspase 8*-mediated proteolytic activation of the normally inert *BH3*-only protein BID (Figure 10; Zhang et al., 2007, 2017; Fan et al., 2014).

Human periodontal ligament fibroblast cells (hPDLFCs) are one of several cell types involved in periodontal tissue regeneration (Son et al., 2019). In addition, few studies have been published on the effect of EPSs on the viability of hPDLFCs. In conclusion, EPS_5_ derived from *L. delbrueckii* ssp. *bulgaricus* DSM 20081^T^ exhibits a range of bioactivities including antioxidant, antitumor, and proliferative properties, thereby establishing it as a possible natural agent for the development of novel periodontal healing and regeneration strategies. Furthermore, because of its extraordinary cytotoxic effect at the IC_50_ value in cancer cells, EPS_5_ could be used in medical research as a potential antitumor agent. As a result, the potential for EPS to replace chemotherapeutic medications in the future must be considered alongside their safety and public health issues. Overall, the LAB EPS has significant therapeutic potential, most notably in the antioxidant, antitumor, and periodontal regeneration fields. However, additional research is necessary to fully understand EPS_5_’s mechanism of action in periodontal healing and tissue restoration.

## Data Availability Statement

The original contributions presented in the study are included in the article/Supplementary Material, further inquiries can be directed to the corresponding author/s.

## Ethics Statement

The studies involving human participants were reviewed and approved by College of Dentistry, Taif University, Taif, Saudi Arabia, Dentistry Clinics Hospitals Ethical Committee Centers. The patients/participants provided their written informed consent to participate in this study.

## Author Contributions

MK: conceptualization, methodology, formal analysis, data curation, and writing—review and editing. FS: conceptualization, validation, and visualization. LA-M and AA: methodology and investigation. TA: methodology and writing—review and editing. SA: statistical analysis, formal analysis, data curation, and writing—review and editing. All authors contributed to the article and approved the submitted version.

## Conflict of Interest

The authors declare that the research was conducted in the absence of any commercial or financial relationships that could be construed as a potential conflict of interest.

## Publisher’s Note

All claims expressed in this article are solely those of the authors and do not necessarily represent those of their affiliated organizations, or those of the publisher, the editors and the reviewers. Any product that may be evaluated in this article, or claim that may be made by its manufacturer, is not guaranteed or endorsed by the publisher.
