# Supplementary material for: Exploring the Therapeutic Potentials of Exopolysaccharides Derived From Lactic Acid Bacteria and Bifidobacteria: Antioxidant, Antitumor, and Periodontal Regeneration
Source: Front Microbiol. 2022 Apr 25;13:803688. doi: 10.3389/fmicb.2022.803688 (PMC9082500; doi:10.3389/fmicb.2022.803688)
Supplement: Supplementary file 1 [file Table_1.DOCX]

**Suppl 1.** Primers for real-time PCR

| Gene | Sequence |
| --- | --- |
| *BCL2*-F  *BCL2*-R | 5'-CGACTTCGCCGAGATGTCCAGCCAG-3'  5'-ACTTGTGGCCCAGATAGGCACCCAG-3' |
| *MCL1*-F  *MCL1*-R | 5′-GGACACAAAGCCAATGGGCAGGT-3′  5′-GCAAAAGCCAGCAGCACATTCCTGA-3 |
| *BAX*-F  *BAX*-R | 5'-AGGGTTTCATCCAGGATCGAGCAG-3'  5'-ATCTTCTTCCAGATGGT GAGCGAG-3' |
| *Caspase3*-F  *Caspase3*-R | 5'-TTAATAAAGGTATCCATGGAGAACACT -3'  5'-TTAGTGATAAAAATAGAGTTCTTTTGTGAG-3' |
| *Caspase8*-F  *Caspase8*-R | 5′-GGACAGGAATGGAACACACTTG-3′  5′-CCATGAGTTGGTAGATTTTCAAAATC-3′ |
| *Vementin* -F  *Vementin*-R | 5′-GACAATGCGTCTCTGGCACGTCTT-3′  5′-TCCTCCGCCTCCTGCAGGTTCTT-3′ |
| *P53*-F  *P53*-R | 5'-TAACAGTTCCTGCATGGGCGGC-3'  5'-AGGACAGGCACAAACACGCACC-3' |
| *GAPDH*-F *GAPDH*-R | 5'-TGCCCTCAACGACCACTTTG-3'  5'-TACTCCTTGGAGGCCATGTG-3' |
